# Targeting *Candida albicans* in dual-species biofilms with antifungal treatment reduces *Staphylococcus aureus* and MRSA *in vitro*

**DOI:** 10.1371/journal.pone.0249547

**Published:** 2021-04-08

**Authors:** Yu Luo, Daniel F. McAuley, Catherine R. Fulton, Joana Sá Pessoa, Ronan McMullan, Fionnuala T. Lundy

**Affiliations:** 1 Wellcome-Wolfson Institute for Experimental Medicine, School of Medicine, Dentistry and Biomedical Sciences, Queen’s University Belfast, Belfast, United Kingdom; 2 Belfast Health & Social Care Trust, Regional Intensive Care Unit, Royal Victoria Hospital, Belfast, United Kingdom; University of Bristol, UNITED KINGDOM

## Abstract

Polymicrobial biofilms consisting of fungi and bacteria are frequently formed on endotracheal tubes and may contribute to development of ventilator associated pneumonia (VAP) in critically ill patients. This study aimed to determine the role of early *Candida albicans* biofilms in supporting dual-species (dual-kingdom) biofilm formation with respiratory pathogens *in vitro*, and investigated the effect of targeted antifungal treatment on bacterial cells within the biofilms. Dual-species biofilm formation between *C*. *albicans* and three respiratory pathogens commonly associated with VAP (*Pseudomonas aeruginosa*, *Escherichia coli and Staphylococcus aureus*) was studied using quantitative PCR. It was shown that early *C*. *albicans* biofilms enhanced the numbers of *E*. *coli* and *S*. *aureus* (including methicillin resistant *S*. *aureus*; MRSA) but not *P*. *aeruginosa* within dual-species biofilms. Transwell assays demonstrated that contact with *C*. *albicans* was required for the increased bacterial cell numbers observed. Total Internal Reflection Fluorescence microscopy showed that both wild type and hyphal-deficient *C*. *albicans* provided a scaffold for initial bacterial adhesion in dual species biofilms. qPCR results suggested that further maturation of the dual-species biofilm significantly increased bacterial cell numbers, except in the case of *E*.*coli* with hyphal-deficient *C*. *albicans* (*Ca_gcn5*Δ/Δ). A targeted preventative approach with liposomal amphotericin (AmBisome^®^) resulted in significantly decreased numbers of *S*. *aureus* in dual-species biofilms, as determined by propidium monoazide-modified qPCR. Similar results were observed when dual-species biofilms consisting of clinical isolates of *C*. *albicans* and MRSA were treated with liposomal amphotericin. However, reductions in *E*. *coli* numbers were not observed following liposomal amphotericin treatment. We conclude that early *C*. *albicans* biofilms have a key supporting role in dual-species biofilms by enhancing bacterial cell numbers during biofilm maturation. In the setting of increasing antibiotic resistance, an important and unexpected consequence of antifungal treatment of dual-species biofilms, is the additional benefit of decreased growth of multi-drug resistant bacteria such as MRSA, which could represent a novel future preventive strategy.

## Introduction

Biofilm formation is a phenotype of many microorganisms, linked to the ability to survive in a hostile host environment [1]. Biofilm-related infections are considered a significant and increasingly prevalent source of morbidity and mortality within the healthcare system and thus much research effort is required towards aiding their prevention and management [2]. Indeed the polymicrobial nature of biofilms associated with infections such as cystic fibrosis [3,4] and chronic wounds [5] is increasingly recognised as an important contributor to disease pathogenesis. Despite our knowledge that biofilms *in vivo* generally contain multiple species encased within an extracellular polymeric matrix, much remains to be learned about the development of mixed species biofilms and the beneficial, parasitic or antagonistic interactions that exist between the microorganisms within them [6–9].

In-dwelling medical devices, many of which are used in critically ill patients, have also been well documented to support polymicrobial biofilm growth [10,11], leading to infections ranging from catheter-related urinary tract infections [10], to ventilator associated pneumonia (VAP) [11,12]. Biofilm colonisation of the endotracheal tube in VAP patients has been reported to occur rapidly after intubation [13], with biofilms acting as reservoirs for pathogenic bacteria [14]. Indeed it has been shown that there is microbiological continuity between airway colonization, biofilm formation and VAP development [15]. Emerging technologies to detect pathogens associated with VAP have identified a broad range of Gram positive and Gram negative bacteria including, *Staphylococcus aureus*, *Escherichia coli and Pseudomonas aeruginosa*, amongst the common bacterial pathogens associated with VAP [16]. Of particular interest is the finding that *Candida albicans* [17], is often present in endotracheal tubes [11], and is associated with an increased risk of VAP (although not as the pathogen causing VAP), as well as prolonged intensive care unit and hospital stays [18]. Given that oral carriage of Candida is reported in up to 40–60% of the population [19,20], it is plausible that *C*. *albicans* could adhere to endotracheal tubes on their insertion through the mouth. Thus, establishment of an early *C*. *albicans* biofilm could facilitate respiratory pathogen integration, leading to subsequent polymicrobial biofilm formation and ultimately drive the development of VAP.

An emerging interest in interactions between fungi and bacteria has highlighted the complexity of fungal-bacterial interactions in polymicrobial and dual-kingdom biofilms, particularly those involving dimorphic fungi. Polymicrobial biofilms are notoriously difficult to eradicate and tend to be recalcitrant to both antimicrobials and host defences [21]. It has been suggested that the morphological plasticity of *C*. *albicans* between yeast and hyphal forms has a major influence on its virulence [22] and that the hyphal form of *C*. *albicans* could provide architecture to the developing biofilm [8,23].

In this study, we investigated dual-species biofilms between *C*. *albicans* and three bacterial species with a view to determining if *C*. *albicans* could enhance respiratory bacterial pathogen numbers in dual-species biofilms, using qPCR for bacterial enumeration. We used a biofilm model in which *C*. *albicans* was inoculated first, to develop an early biofilm before the addition of the bacterial species. We also investigated whether contact was needed between the early *C*. *albicans* biofilm and bacterial cells to enhance bacterial cell numbers. Using Total Internal Reflection Fluorescence (TIRF) microscopy, we imaged bacterial attachment to wild type and hyphal-deficient *C*. *albicans* and by qPCR determined bacterial cell numbers during dual-species biofilm maturation. Furthermore, we examined whether a targeted preventative approach with an antifungal could reduce bacterial cell numbers in dual-species biofilms. The current work has potential clinical implications as it could open up new preventive and therapeutic targets for the management of polymicrobial biofilms infections such as VAP, with the possibility of decreasing bacterial numbers of *S*. *aureus* and MRSA without antibiotics, thereby lowering the risk of developing antibiotic resistance.

## Materials and methods

### Micro-organism strains and growth conditions

*C*. *albicans* (NCTC 3179) was sub-cultured aerobically on Sabouraud agar plates and propagated in yeast peptone dextrose (YPD) broth (US Biological). *P*. *aeruginosa* (ATCC 27853), *E*. *coli* (ATCC 29522), *S*. *aureus* (NCTC 6571) and methicillin resistant *S*. *aureus* (MRSA; MRSA 4D) were grown on blood agar plates and propagated in brain heart infusion (BHI) broth. The laboratory strain (wild type) of *C*. *albicans* (NCTC 3179) was used in all experiments, except where otherwise stated, such as dual-species biofilm treatment with liposomal amphotericin, in which a clinical isolate of *C*. *albicans* (CA 239SB) was also studied. In experiments on dual-species biofilm maturation, a *C*. *albicans* hyphal-deficient mutant (*Ca_gcn5*Δ/Δ) [24] (gift from Prof Karl Kuchner, Medical University of Vienna) was studied along with the laboratory strain of *C*. *albicans*. The clinical isolate and hyphal-deficient mutant of *C*. *albicans* were sub-cultured as described for the laboratory strain.

### Preparation of single species bacterial biofilms

Overnight cultures (18 hours) of *P*. *aeruginosa*, *E*. *coli*, *S*. *aureus* and MRSA were resuspended in BHI to yield inocula of 5.0 x 10^6^ cells/ml. A total volume of 100 μl of each inoculum in BHI was added to microtitre plate wells (Thermo Fisher Scientific, Roskilde, Denmark), and the plates were incubated at 37°C for 4 hours to allow initial biofilm formation under static growth conditions. Wells were washed carefully, three times with PBS, to remove planktonic cells and the biofilms incubated with 100 μl fresh broth for a further 24 hours to allow biofilm maturation. Biofilms were then washed to remove planktonic cells and quantified by qPCR as outlined below. Single species bacterial biofilms were grown in BHI throughout (4 hours for initial biofilm formation, plus an additional 24 hours for early biofilm maturation).

### Preparation of dual-species biofilms

A schematic outline of dual-species biofilm formation, treatment and quantification is shown in S1 Fig. Overnight cultures (18 hours) of *C*. *albicans* were washed with phosphate-buffered saline (PBS) and resuspended in a modified Roswell Park Memorial (RPMI) medium (RPMI-1640; Sigma-Aldrich, St Louis, USA), referred to subsequently as RPMI, to yield an inoculum of 1.0 x 10^6^ cells/ml [25]. The *C*. *albicans* inoculum (100 μl) was added to microtitre plate wells (Thermo Fisher Scientific, Roskilde, Denmark) and incubated under static growth conditions at 37°C for 4 hours to allow initial biofilm formation. The biofilm was washed three times with PBS to remove planktonic *C*. *albicans* cells and spent RPMI, prior to inoculation with 100 μl of *P*. *aeruginosa*, *E*. *coli*, *S*. *aureus*, or MRSA (5 x 10^6^ cells/ml) in BHI. Bacteria were allowed to adhere to the initial *C*. *albicans* biofilms for 4 hours to facilitate dual-species biofilm formation. Following washing to remove planktonic cells and spent media as outlined above, biofilms were incubated in BHI for a further 24 hours to allow dual-species biofilm maturation. Wells were then washed with PBS as outlined above and the biofilms were quantified by qPCR (S1A Fig). Single species bacterial biofilms served as controls.

In selected experiments, to further investigate the role of *C*. *albicans* in enhancing bacterial cell numbers during biofilm maturation, we prepared dual-species biofilms as outlined above with wild type *C*. *albicans or C*. *albicans gcn5*Δ/Δ hyphal-deficient mutant. We then quantified bacterial numbers after initial adhesion (4 hours), or following a combined initial adhesion period (4 hours) and further maturation period (24 hours). Quantification of bacterial cell numbers was undertaken by qPCR as described below.

### Bacterial cell quantification in biofilms by qPCR

To quantify cell numbers in single species or dual-species biofilms, the biofilms were detached from microtitre wells into 100 μl of broth by sonicating for 5 min in an ultrasonic bath (Dawe, Middlesex, UK). Any remaining cells were then collected into a further 100 μl broth. DNA was extracted using the microLYSIS^®^-Plus kit (Microzone, Haywards Heath, UK) as per the manufacturer’s instructions and individual monoplex qPCR assays were performed using an Mx3005P qPCR System (Agilent Technologies, California USA) as detailed in S1–S5 Tables (Supporting information). The candidate genes selected were expressed by the specific micro-organisms used in this study and the primers employed for their quantification had been designed and published previously: Primers for *P*. *aeruginosa* were against the *oprL* gene [26]; Primers for *E*. *coli* quantification were against the 16S rRNA gene [27]; Primers for *S*. *aureus* quantification were against the Panton-Valentive leucocidin (PVL) gene [28]. The *S*. *aureus* strains NCTC 6571 and MRSA 4D used in this work both harboured the PVL gene.

### Generation of standard curves for qPCR

To allow for quantification of bacterial cell numbers, DNA standards were prepared by extraction of DNA from planktonic organisms using the microLYSIS®-Plus kit and purified using the DNeasy kit (Qiagen, Manchester, UK). DNA standards, as previously described by us [29], were used in all qPCR assays to generate standard curves from which the numbers of organisms within the biofilms could be determined. Cell numbers within the biofilm were thus generated from and were equivalent to cell numbers from planktonic cultures used in the standard curves.

### Transwell assays for determining the influence of contact with *C*. *albicans* biofilms on *E*. *coli*, *S*. *aureus* and MRSA cell numbers

To determine whether contact with early *C*. *albicans* biofilms was required to enhance respiratory pathogen cell numbers, Transwell assays were employed in which *C*. *albicans* biofilms grown in the upper chamber, were physically separated from bacteria (*E*. *coli*, *S*. *aureus*, or MRSA) in the lower chamber. A 1 ml inoculum of *C*. *albicans* (1.0 x 10^6^ cells/ml in RPMI) was added to the upper chamber (Costar Transwell 12 mm, 0.4 μm Polyester Membrane, Corning NY) and incubated for 4 hours to allow initial *C*. *albicans* biofilm formation. The *C*. *albicans* biofilm was washed three times with PBS and the lower chamber was inoculated with 2 ml of bacteria (5 x 10^6^ cells/ml in BHI) before replacing the *C*. *albicans*-coated upper chamber containing 1 ml BHI. Transwells were incubated for a further 4 hours before washing both chambers three times with PBS and adding fresh BHI (1 ml upper chamber and 2 ml lower chamber). Biofilms were incubated for a further 24 hours to allow maturation before bacterial biofilm quantification by qPCR as outlined above. For control experiments, the upper chamber was not inoculated with *C*. *albicans*, but all other steps were undertaken as outlined above. No Transwell experiments were undertaken with *P*. *aeruginosa*, as cell numbers of *P*. *aeruginosa* did not increase in the dual-species *C*. *albicans-P*. *aeruginosa* biofilms that we studied.

### Electroporation of reporter plasmids into *E*.*coli*, *S*. *aureus* and MRSA

*S*. *aureus* (NCTC 6571) and MRSA (4D) were grown in BHI overnight, centrifuged for 10 minutes, resuspended in 300 mM sucrose (to 1/10 volume) and 100 μL of this mixture was electroporated (25 μF, 200 Ω, 2.5 kV) with 100 ng of a derivative of pCN47 containing the phyper promoter and GFP [30] (a gift from Prof Iñigo Lasa, Universidad Pública de Navarra). *E*. *coli* (ATCC 29522) was prepared in the same manner and electroporated (25 μF, 200 Ω, 2.5 kV) with 100 ng of pUC18T-mini-Tn7T-Apr-mCherry [31] (a gift from Dr Ayush Kumar, University of Manitoba).

### Dual-species biofilm preparation for TIRF microscopy

Overnight cultures of *C*. *albicans* and mCherry-labelled *E*. *coli*, GFP-labelled *S*. *aureus*, or GFP-labelled MRSA were prepared as outlined above and biofilms were formed in μ-Slide 8 well ibiTreat chamber slides (ibidi, Germany) by inoculating wells with 100 μl *C*. *albicans* laboratory strain or *C*. *albicans gcn5*Δ/Δ hyphal-deficient mutant (1.0 x 10^6^ cells/ml in RPMI). Wells were incubated for 4 hours to allow initial *C*. *albicans* biofilm formation. The biofilm was washed with PBS to remove planktonic cells and RPMI, prior to inoculation with 100 μl mCherry- or GFP-labelled bacteria (5.0 x 10^6^ cells/ml in BHI). Bacteria were allowed to adhere to the *C*. *albicans* biofilms for 4 hours to facilitate initial dual-species biofilm formation. Images were acquired by TIRF microscopy (Leica UK) in the epifluorescence mode using a Leica EL6000 external light source for fluorescent images and LED lamp for bright field images. Fluorescent and bright field images were overlaid using LAS-X software (Leica Application suite).

### Dual-species biofilm treatment with liposomal amphotericin and subsequent quantification by PMA-qPCR

To determine the effect of antifungal treatment on bacterial numbers in dual species biofilms, liposomal amphotericin (1 μg/ml; reflecting the clinical breakpoint recommended by EUCAST) was added to *C*. *albicans* (NCTC 3179) or clinical isolate of *C*. *albicans* (CA 239SB) inoculum preparations, before addition to the wells of the microtitre plates (S1B Fig). The remaining steps for dual-species biofilm were as outlined above, the only exception being that after the last washing step at each stage of the protocol, 1 μg/ml liposomal amphotericin was added to the BHI media (S1B Fig). Following biofilm treatment with liposomal amphotericin, it was important to quantify only living cells within the biofilm and thus a PMA (Biotium Inc., California, USA) qPCR protocol was employed as previously described by us [29]. Briefly, following biofilm detachment from microtitre plates, as outlined above, the DNA-intercalating agent PMA (200 μM) was added to each tube and incubated at 37°C for 5 min, prior to photo-activation with a broad-spectrum LED flood light [32]. PMA binds to DNA from dead cells and prevent its amplification by qPCR, thereby allowing quantification of DNA from living cells only. DNA was then extracted and quantified by qPCR using an Mx3005P qPCR System (Agilent Technologies, California USA) with the reaction conditions outlined in S1–S5 Tables (Supporting information).

### Statistical analysis

Datasets were analysed by nonparametric Mann-Whitney statistical tests or Kruskal-Wallis tests for multiple comparisons, as detailed in figure legends. Data from a total of three independent experiments were analysed for each dataset. Statistical analysis and graphing of data was performed using GraphPad Prism version 8 for Windows. A p value of <0.05 was considered statistically significant.

## Results

### *C*. *albicans* early biofilms enhance cell numbers of selected respiratory pathogens

We initially studied whether the presence of early biofilms of *C*. *albicans* could enhance cell numbers of *P*. *aeruginosa*, *E*. *coli*, *S*. *aureus* or MRSA (S2 Fig). In *C*. *albicans*-*P*. *aeruginosa* dual-species biofilms, *P*. *aeruginosa* cell numbers (4.3x10^6^) were not significantly different to those in axenic *P*. *aeruginosa* biofilms (3.4x10^6^) (S2A Fig) and it was not studied further. However, numbers of *E*. *coli* (8.9x10^6^) were significantly increased in the presence of early *C*. *albicans* biofilms compared with axenic *E*. *coli* biofilms (3.0x10^6^) (S2B Fig). Likewise, *S*. *aureus* numbers were significantly increased in dual-species (2.6x10^7^) compared with axenic biofilms (1.2x10^7^) (S2C Fig). To demonstrate that results obtained with laboratory strains were also applicable to clinical isolates we showed that cell numbers of a clinical isolate of MRSA were significantly increased when grown in the presence of a clinical isolate of *C*. *albicans* (2.4x10^7^) compared with axenic MRSA biofilms (1.0x10^6^) (S2D Fig).

### Contact with *C*. *albicans* early biofilms is required to enhance cell numbers of respiratory pathogens

**Fig 1:**
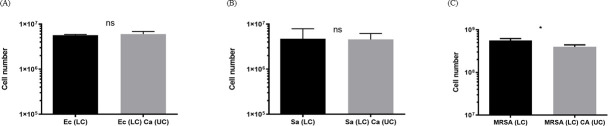
Increased bacterial cell numbers in dual species biofilms is contact dependent with early *C*. *albicans* biofilm. A two chamber Transwell assay was used to determine whether contact between early *C*. *albicans* biofilms was required for enhanced bacterial biofilm cell numbers. (A) The number of *E*. *coli*, (B) *S*. *aureus* and (C) MRSA cells in the lower chamber biofilm was quantified by qPCR. Micro-organisms in the two chambers were physically separated from each other but shared the same medium. (Mann Whitney statistical analysis; 3 independent experiments. ns: p > 0.05, *: p<0.05, error bars SD).

To determine whether contact between *C*. *albicans* and bacteria was required for the increased bacterial cell numbers observed, *C*. *albicans* early biofilms were separated from respiratory pathogenic bacteria in Transwell assay experiments. In the absence of contact between *C*. *albicans* and bacteria, no increase in *E*.*coli*, *S*. *aureus* or MRSA cell numbers was observed (Fig 1) and indeed MRSA numbers were shown to decrease.

### Dual species biofilm formation with *C*. *albicans* wild type and hyphal-deficient mutant

**Fig 2:**
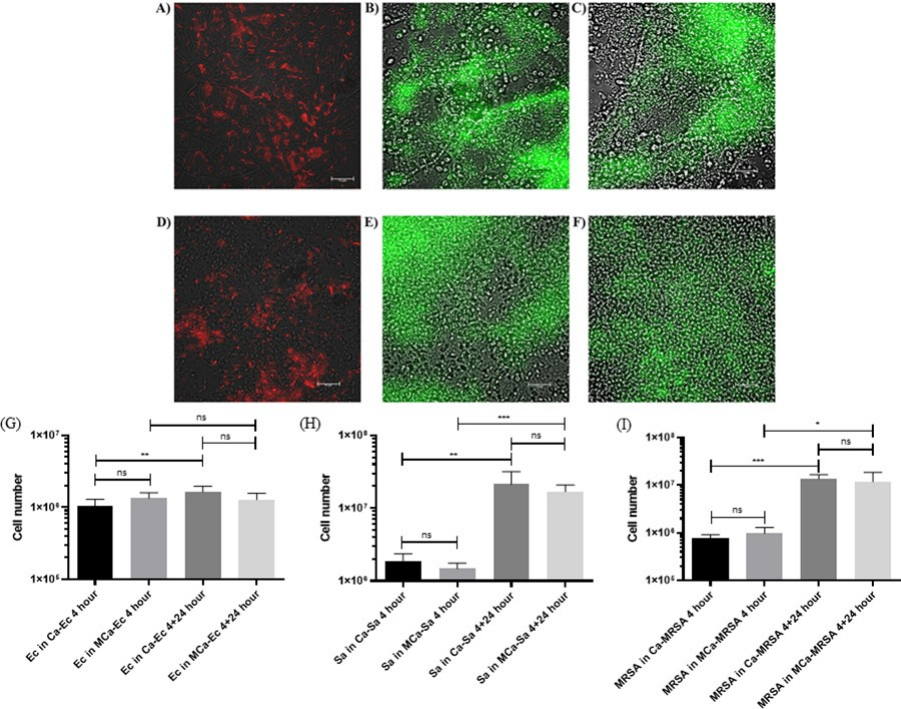
Dual-species biofilm formation with *C*. *albicans* wild type or hyphal-deficient mutant investigated by TIRF fluorescent microscopy and qPCR. Dual-species biofilms of (A) *C*. *albicans* NCTC 3179 and mCherry-*E*.*coli*, (B) *C*. *albicans* NCTC 3179 and GFP-*S*. *aureus*, (C) *C*. *albicans* NCTC 3179 and GFP-MRSA. Dual-species biofilms of (D) *C*. *albicans* hyphal-deficient mutant (*Ca_gcn5*Δ/Δ) and mCherry-*E*.*coli*, (E) *C*. *albicans* hyphal-deficient mutant (*Ca_gcn5*Δ/Δ) and GFP-*S*. *aureus*, (F) *C*. *albicans* hyphal-deficient mutant (*Ca_gcn5*Δ/Δ) and GFP-MRSA. Scale bar 15 μm. Dual species biofilms containing *C*. *albicans* wild type (NCTC 3179) (Ca) or hyphal-deficient mutant (*Ca_gcn5*Δ/Δ) (MCa) were investigated by qPCR to determine (G) *E*. *coli* (Ec), (H) *S*. *aureus* (Sa) and (I) MRSA numbers in dual-species biofilms after initial adhesion to *C*. *albicans* (4 hours), or following a combined initial adhesion period of 4 hours and a further maturation period of 24 hours. (Kruskal-Wallis statistical analysis; 3 independent experiments. ns: p > 0.05, *: p<0.05, ** p<0.01 ***: p < 0.01, error bars SD).

To confirm the presence of *C*. *albicans* biofilms after 4 hours initial biofilm formation, we demonstrated the presence of extracellular polymeric matrix, using SYPRO™ Ruby Biofilm matrix stain (S3 Fig). Having established the presence of any early biofilm, we wanted to determine if hyphae had a role in enhancing bacterial numbers following inoculation of respiratory pathogens on the pre-formed *C*. *albicans* biofilm. TIRF microscopy of wild type and hyphal-deficient *C*. *albicans* showed attachment of *E*. *coli*, *S*. *aureus* and MRSA following 4 hours initial adhesion (Fig 2A–2F). To further elucidate the role of *C*. *albicans* hyphae, we quantified cell numbers of *E*. *coli*, *S*. *aureus* and MRSA after 4 hours initial adhesion to wild type or hyphal-deficient mutant *C*. *albicans* biofilms and then after an additional 24 hours (corresponding to further maturation of the dual-species biofilms). Following the initial bacterial adhesion period to the pre-formed *C*. *albicans* biofilms, no significant differences in bacterial numbers attached to wild type or hyphal-deficient mutant *C*. *albicans* were observed. Following dual species biofilm maturation for a further 24 hours *E*. *coli*, *S*. *aureus* and MRSA numbers increase significantly in biofilms with wild type *C*. *albicans*. However, during the maturation phase with the hyphal-deficient *C*. *albicans* only *S*. *aureus* and MRSA increased significantly, with *E*. *coli* numbers failing to do so (Fig 2G–2I).

### Targeting *C*. *albicans* with liposomal amphotericin in dual-species biofilms decreased *S*. *aureus* and MRSA cell numbers

**Fig 3:**
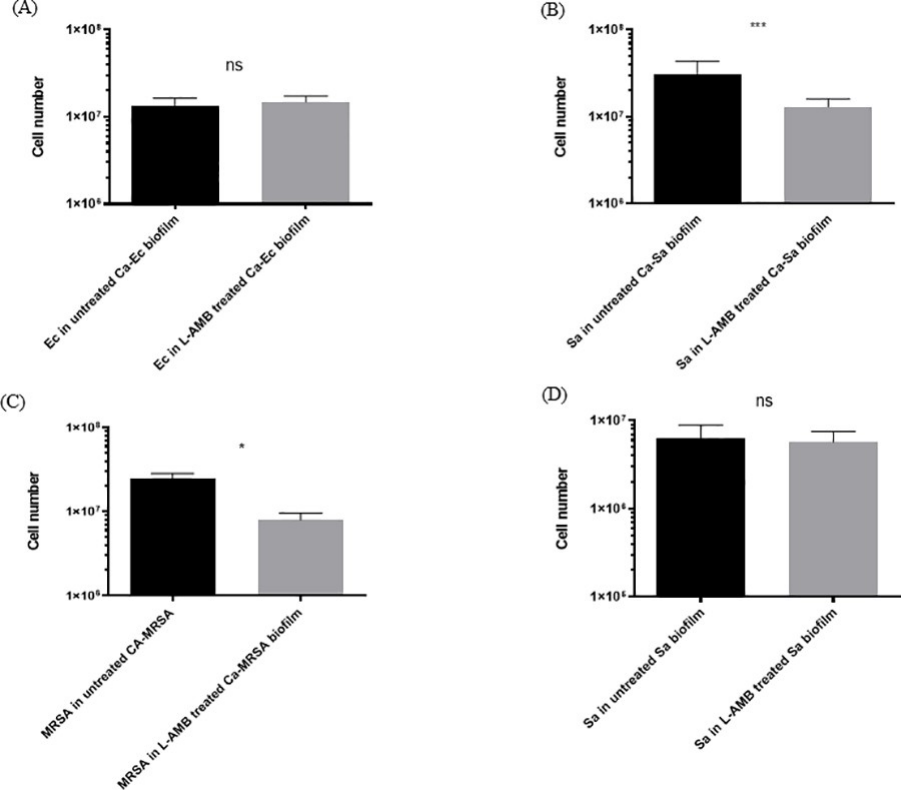
PMA-qPCR quantification of *E*.*coli*, *S*. *aureus* and MRSA in liposomal amphotericin (AmBisome®) treated dual-species and axenic *S*. *aureus* biofilms. (A) Cell numbers of *E*. *coli* (Ec) and (B) *S*. *aureus* (Sa) in untreated and liposomal amphotericin (L-AMB) treated dual-species biofilms grown with *C*. *albicans* laboratory strain (NCTC 3179). (C) Cell numbers of MRSA (clinical isolate) in untreated and liposomal amphotericin (L-AMB) treated dual-species biofilms grown with *C*. *albicans* clinical isolate. (D) Cell numbers of *S*. *aureus* (Sa) in untreated and L-AMB treated axenic biofilms. (Mann-Whitney statistical analysis, 3 independent experiments ns: p > 0.05; *: p < 0.05, ***: p < 0.001, error bars SD).

In view of our results showing that *C*. *albicans* early biofilms enhanced *E*.*coli*, *S*. *aureus* and MRSA numbers, we were prompted to test a novel approach aimed at decreasing pathogenic bacterial cell numbers by targeting the architecturally supporting micro-organism, *C*. *albicans*, with the antifungal drug liposomal amphotericin. Following treatment of dual-species with liposomal amphotericin, no significant reduction *in E*.*coli* cell numbers was observed (Fig 3A). However, liposomal amphotericin treatment of dual species *C*. *albicans*-*S*. *aureus* biofilms resulted in significant reduction of *S*. *aureus* (Fig 3B) cell numbers. Moreover, in dual-species biofilms containing clinical isolates of *C*. *albicans* and MRSA, liposomal amphotericin treatment significantly reduced MRSA numbers (Fig 3C). Cell numbers in axenic *S*. *aureus* biofilms were not significantly altered by liposomal amphotericin treatment (Fig 3D), suggesting it did not have a direct effect on *S*. *aureus* within the biofilms.

## Discussion

*C*. *albicans* is often overlooked as a bystander in polymicrobial biofilm-associated infections. However, using qPCR to determine bacterial cell numbers within dual-species biofilms [29] we showed that *C*. *albicans* early biofilms enhanced cell numbers of *S*. *aureus*, MRSA and *E*. *coli*, but not *P*. *aeruginosa*. Our results, which focus on quantifying bacterial cell numbers, agree with previous studies in which *C*. *albicans* was shown to enhance *S*. *aureus* dual-species biofilm formation in the presence of serum [33,34] and that an antagonistic relationship exists between *P*. *aeruginosa* and *C*. *albicans* [35,36].

Using a Transwell assay, we demonstrated that contact between early *C*. *albicans* biofilms and *E*. *coli*, *S*. *aureus* or MRSA was required to enhance bacterial cell numbers. Previously, using a Transwell assay Harriott & Noverr [33] showed contact requirement when *C*. *albicans* and *S*. *aureus* were inoculated simultaneously in Transwell upper and lower chambers respectively. In our Transwell assay (as in our biofilm model) *C*. *albicans* was inoculated first (upper chamber) to allow early biofilm development before bacterial inoculation (lower chamber). Despite the presence of an early *C*. *albicans* biofilm, soluble factors from the early biofilm did not support increased numbers of bacteria. Our results therefore concur that contact is required between *C*. *albicans* and *S*. *aureus* [33] and also between *C*. *albicans* and *E*. *coli* or MRSA, even when an early *C*. *albicans* biofilm is present. The decreased cell numbers of MRSA observed in the Transwell assay results, highlight that additional factors, such as the production of farnesol by *C*. *albicans* [37], which is recognised to have inhibitory effects against MRSA [38], may be more evident in the absence of direct fungal-bacterial contact.

In the presence of direct contact, the architecture of *C*. *albicans* has been reported to have an important role in polymicrobial biofilms [8,23,34,39,40]. *C*. *albicans* adhesion proteins such as the agglutinin-like sequence 3 protein (Als3p) [41,42], as well as Staphylococcal adhesins such as fibronectin binding protein B (FnpB), *S*. *aureus* surface protein F (SasF) or a putative N-acelymuramoul-1-alanine amidase (Atl) [43,44] may contribute to physical interactions in dual-species biofilm formation. Although our results do not appear to concur with previous work on the importance of the hyphal adhesion protein Als3p in *S*. *aureus* adhesion, multiple additional *C*. *albicans* adhesins are likely to have roles in biofilm formation [45]. Moreover, yeast wall protein 1 (Ywp1), a protein downregulated during filamentation [46] and previously reported to regulate dispersion in *C*. *albicans*, has recently been shown to function in maintaining adhesion following initial attachment [45]. Thus, biofilm formation is likely to involve multiple adhesins, with complex temporal and spatial roles in the initial attachment and biofilm maturation stages. Furthermore, although mediators such as prostaglandin E2 (PGE2) have been reported to enhance *C*. *albicans*-*S*. *aureus* biofilm formation [47], the concentration of PGE2 produced by a 24 hour culture of *C*. *albicans* has been shown to be insufficient to do so [47]. Thus, while soluble factors may enhance bacterial numbers in maturing dual-species biofilms, our results suggest that during the initial stages of dual-species biofilm formation, contact is essential.

TIRF microscopy of wild type or hyphal-deficient mutant *C*. *albicans* showed attachment of *E*. *coli*, *S*. *aureus* and MRSA in early dual-species biofilms. Quantification of bacterial numbers in early and maturing dual-species biofilms showed that hyphal-deficient *C*. *albicans* was capable of significantly enhancing *S*. *aureus* and MRSA, but not *E*. *coli* numbers, suggesting that subtle differences may exist in the ability of the *C*. *albicans* strains to increase bacterial numbers in the maturing biofilms.

It is well recognised that liposomal amphotericin has enhanced efficacy against *C*. *albicans* biofilms [48,49] and as expected, *C*. *albicans* numbers were decreased in all dual species biofilms studied, following liposomal amphotericin treatment (results not shown). Interestingly, we found that liposomal amphotericin treatment of dual-species *C*. *albicans*-*S*. *aureus* or *C*. *albicans*-MRSA biofilms also resulted in a significant reduction in *S*. *aureus* and MRSA cell numbers, despite their being no direct effect of the antifungal on *S*. *aureus* axenic biofilms. Notwithstanding some previous indications of potential synergistic relationships between *C*. *albicans* and *S*. *aureus* [35,50,51], antifungal treatments have not been actively pursued as a potential mechanism to reduce bacterial burden. Several elegant studies on *C*. *albicans* dual-species biofilms have previously reported that fungal cells may modulate the action of antibiotics [33,39,40,52]. However, no studies to date have successfully targeted *C*. *albicans* as a means of reducing bacterial cell numbers in dual-species biofilms. It had previously been suggested that in the absence of a known antibacterial effect of miconazole against *S*. *aureus*, the clinical success of topical treatment with the antifungal, miconazole, could potentially be attributed to direct activity against the *C*. *albicans* biofilm meshwork, which could destabilise *S*. *aureus* colonization [42]. However, more recently, the imidazole antifungal miconazole has indeed been shown to have direct efficacy against *S*. *aureus* [53] and therefore the clinical efficacy of topical miconazole could involve direction antibacterial action. What we show in the current study, which has not been reported previously, is that targeting *C*. *albicans* with liposomal amphotericin in dual-species biofilms reduces *S*. *aureus* and MRSA numbers, without direct antibacterial/off-target effects on *S*. *aureus*.

Interesting, liposomal amphotericin treatment of dual-species *C*. *albicans-E*.*coli* did not reduce *E*. *coli* numbers. Our data on dual-species biofilm development in either wild type or hyphal-deficient *C*. *albicans*, showed that *E*.*coli* numbers failed to increase significantly during biofilm maturation with the hyphal-deficient mutant, suggesting that hyphae may have a more prominent role in dual-species *C*. *albicans-E*.*coli* biofilm formation. With this in mind, it is possible that, following liposomal-treatment, dead hyphae may still be able to provide architectural support for *E*. *coli*, a phenomenon that has been described in other fungal species [54]. Such an effect could potentially contribute to the lack of reduction in *E*. *coli* numbers that we observed following liposomal-treatment of *C*. *albicans-E*.*coli* biofilms.

## Conclusions

In conclusion, we demonstrate that early *C*. *albicans* biofilms facilitated increased numbers of *E*. *coli*, *S*. *aureus* and MRSA in dual-species biofilms via direct contact. Furthermore, treatment of dual-species biofilms with liposomal amphotericin significantly reduced *S*. *aureus* and MRSA cell numbers but not *E*. *coli* numbers. Given the importance of fungal-bacterial biofilms in a wide range of human diseases [55], the identification of *C*. *albicans* as a target micro-organism in polymicrobial biofilm infections may have important clinical consequences. Targeted treatment of early *C*. *albicans* biofilms could be developed to reduce not only fungal but also bacterial burdens in polymicrobial biofilm infections. The controlled release [56,57] of surface-bound antifungal drugs may prove useful in this respect.

## Supporting information

S1 Fig Schematic outline for polymicrobial biofilm formation, treatment and quantification. (A) Polymicrobial biofilm formation and quantification by qPCR. Inoculation of *C*. *albicans* in Roswell Park Memorial Institute (RPMI) broth, followed by inoculation and subsequent growth of *S*. *aureus*, MRSA, *E*. *coli* or *P*. *aeruginosa* in brain heart infusion (BHI) broth. (B) Treatment of polymicrobial biofilms with liposomal amphotericin (1μg/ml; EUCAST clinical breakpoint) and subsequent quantification by propidium monoazide (PMA)-qPCR.
(DOCX)

S2 Fig qPCR quantification of respiratory pathogens in axenic and polymicrobial biofilms (consisting of C. albicans (Ca) and respiratory pathogen). (A) Cell numbers of *P*. *aeruginosa* (Pa). (B) Cell numbers of *E*. *coli* (Ec). (C) Cell numbers of *S*. *aureus* (Sa). (D) Cell numbers of MRSA. All respiratory pathogenic bacteria were detected in axenic and polymicrobial biofilms by qPCR using specific primers. Data from a total of three independent experiments (Mann Whitney; ns: p > 0.05; **: p < 0.01, ***: p < 0.001, error bars SD).
(DOCX)

S3 Fig Confocal fluorescent microscopy of C. albicans 4 hour biofilm. To demonstrate the presence of an early biofilm containing extracellular matrix, chamber slides were stained with SYPRO™ Ruby Biofilm Matrix Stain following 4 hours incubation with *C*. *albicans* (see S1 Methods). Image acquired using a Leica TCS SP8 confocal laser scanning microscope (Leica, UK). To preserve the image data (without modification) images were processed in 3D, using LAS-X software (Leica Application suite), for addition of a 3D scale.
(DOCX)

S1 Table qPCR reaction formulation for S. aureus. (DOCX)

S2 Table qPCR reaction formulation for E. coli. (DOCX)

S3 Table qPCR reaction formulation for P. aeruginosa. (DOCX)

S4 Table qPCR conditions for C. albicans and E. coli (instructions provided with FastSart kit, Roche). (DOCX)

S5 Table qPCR conditions for S. aureus and P. aeruginosa (adapted from instructions provided with Platinum® quantitative PCR SuperMix-UDG). (DOCX)

S1 Methods (DOCX)
