# Supplementary material for: Targeting Candida albicans in dual-species biofilms with antifungal treatment reduces Staphylococcus aureus and MRSA in vitro
Source: PLoS One. 2021 Apr 8;16(4):e0249547. doi: 10.1371/journal.pone.0249547 (PMC8031443; doi:10.1371/journal.pone.0249547)
Supplement: S1 Fig — (A) Polymicrobial biofilm formation and quantification by qPCR. Inoculation of C. albicans in Roswell Park Memorial Institute (RPMI) broth, followed by inoculation and subsequent growth of S. aureus, MRSA, E. coli or P. aeruginosa in brain heart infusion (BHI) broth. (B) Treatment of polymicrobial biofilms with liposomal amphotericin (1μg/ml; EUCAST clinical breakpoint) and subsequent quantification by propidium monoazide (PMA)-qPCR. (DOCX) [file pone.0249547.s001.docx]

**Supplementary Figure 1**


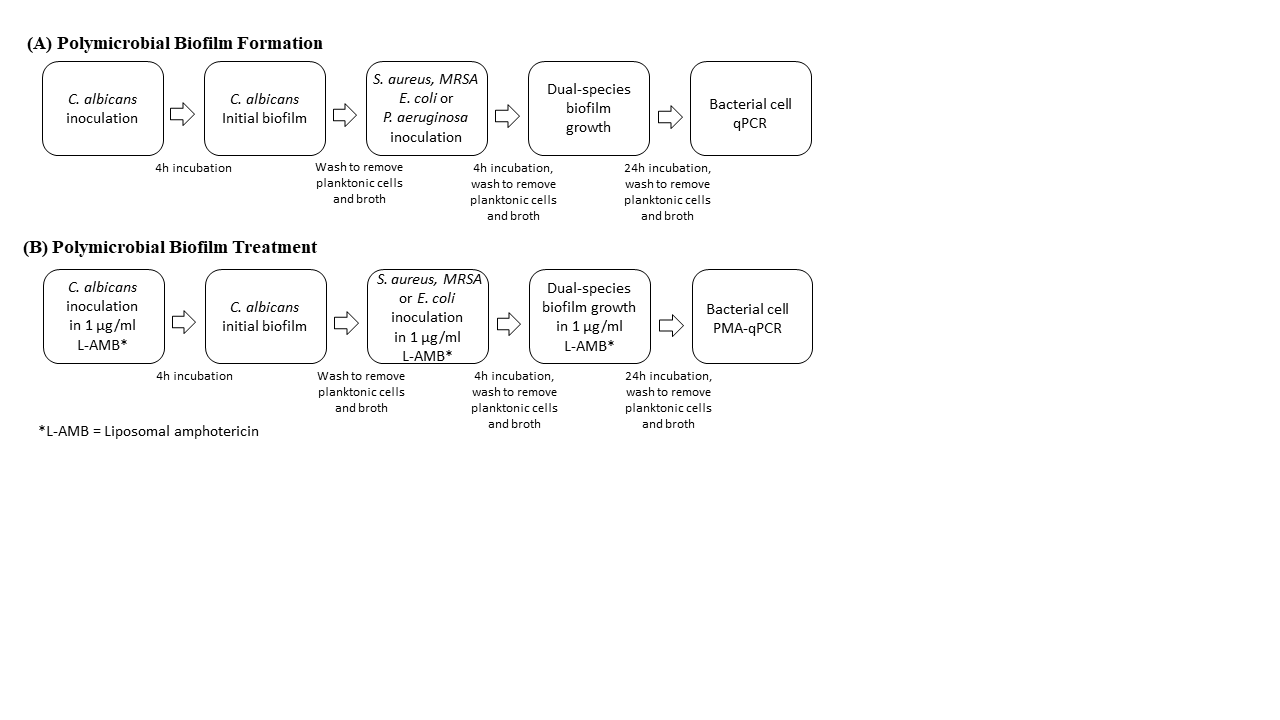


**Supp Fig 1. Schematic outline for polymicrobial biofilm formation, treatment and quantification**.

(A) Polymicrobial biofilm formation and quantification by qPCR. Inoculation of *C. albicans* in Roswell Park Memorial Institute (RPMI) broth, followed by inoculation and subsequent growth of *S. aureus*, MRSA, *E. coli* or *P. aeruginosa* in brain heart infusion (BHI) broth. (B) Treatment of polymicrobial biofilms with liposomal amphotericin (1µg/ml; EUCAST clinical breakpoint) and subsequent quantification by propidium monoazide (PMA)-qPCR.
