# Supplementary material for: Targeting Candida albicans in dual-species biofilms with antifungal treatment reduces Staphylococcus aureus and MRSA in vitro
Source: PLoS One. 2021 Apr 8;16(4):e0249547. doi: 10.1371/journal.pone.0249547 (PMC8031443; doi:10.1371/journal.pone.0249547)
Supplement: S2 Fig — (A) Cell numbers of P. aeruginosa (Pa). (B) Cell numbers of E. coli (Ec). (C) Cell numbers of S. aureus (Sa). (D) Cell numbers of MRSA. All respiratory pathogenic bacteria were detected in axenic and polymicrobial biofilms by qPCR using specific primers. Data from a total of three independent experiments (Mann Whitney; ns: p > 0.05; **: p < 0.01, ***: p < 0.001, error bars SD). (DOCX) [file pone.0249547.s002.docx]

**Supplementary Figure 2**

(A)

(B)


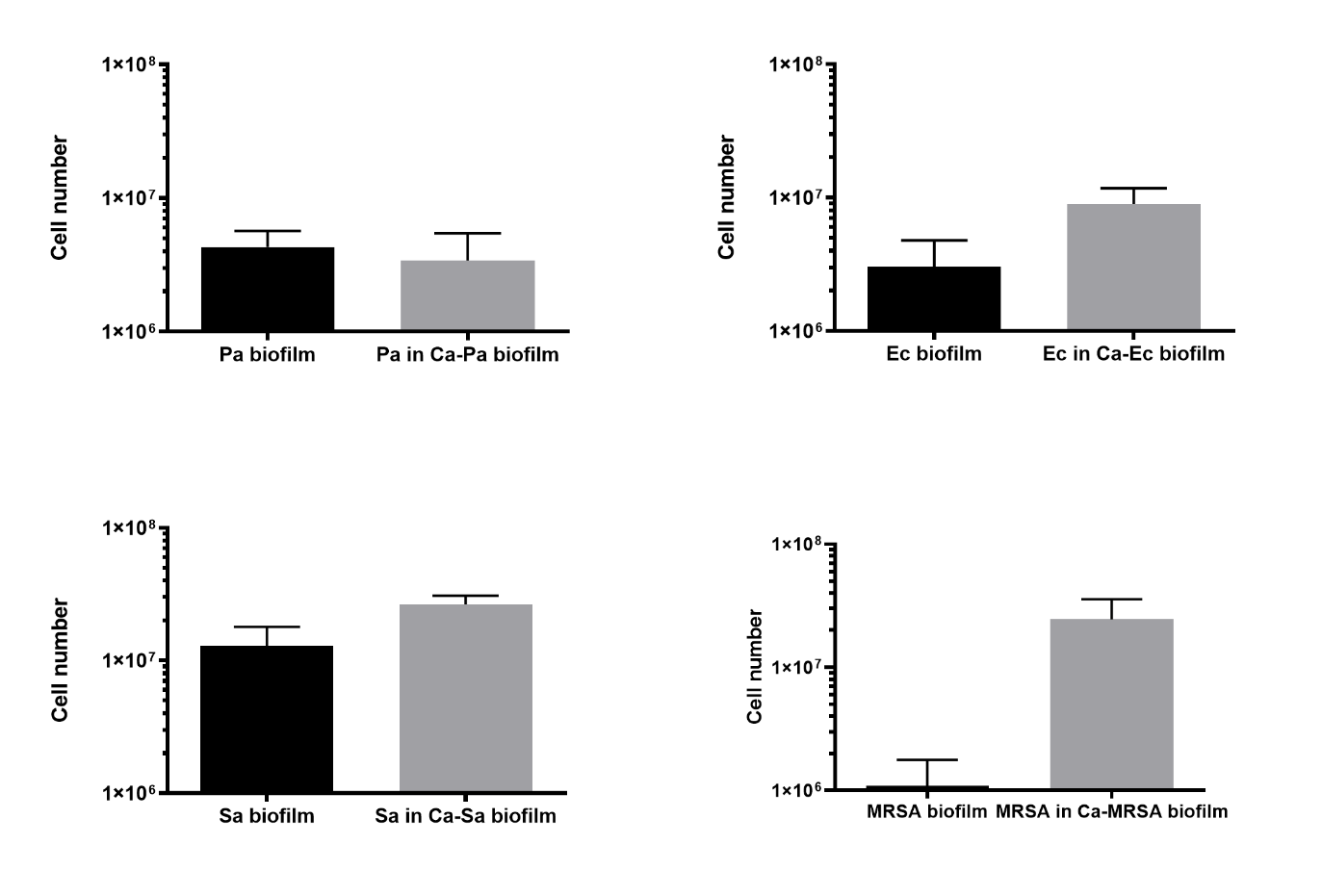


***

***

ns

(D)

(C)

**

**Supp Fig 2. qPCR quantification of respiratory pathogens in axenic and polymicrobial**

**biofilms (consisting of C. *albicans* (Ca) and respiratory pathogen)**.

(A) Cell numbers of *P. aeruginosa* (Pa). (B) Cell numbers of *E. coli* (Ec). (C) Cell numbers

of *S. aureus* (Sa). (D) Cell numbers of MRSA. All respiratory pathogenic bacteria were

detected in axenic and polymicrobial biofilms by qPCR using specific primers. Data from a total of three independent experiments (Mann Whitney; ns: p > 0.05; **: p < 0.01,

***: p < 0.001, error bars SD).
