# Supplementary material for: Targeting Candida albicans in dual-species biofilms with antifungal treatment reduces Staphylococcus aureus and MRSA in vitro
Source: PLoS One. 2021 Apr 8;16(4):e0249547. doi: 10.1371/journal.pone.0249547 (PMC8031443; doi:10.1371/journal.pone.0249547)
Supplement: S3 Fig — To demonstrate the presence of an early biofilm containing extracellular matrix, chamber slides were stained with SYPRO™ Ruby Biofilm Matrix Stain following 4 hours incubation with C. albicans (see S1 Methods). Image acquired using a Leica TCS SP8 confocal laser scanning microscope (Leica, UK). To preserve the image data (without modification) images were processed in 3D, using LAS-X software (Leica Application suite), for addition of a 3D scale. (DOCX) [file pone.0249547.s003.docx]

**Supplementary Figure 3**

**
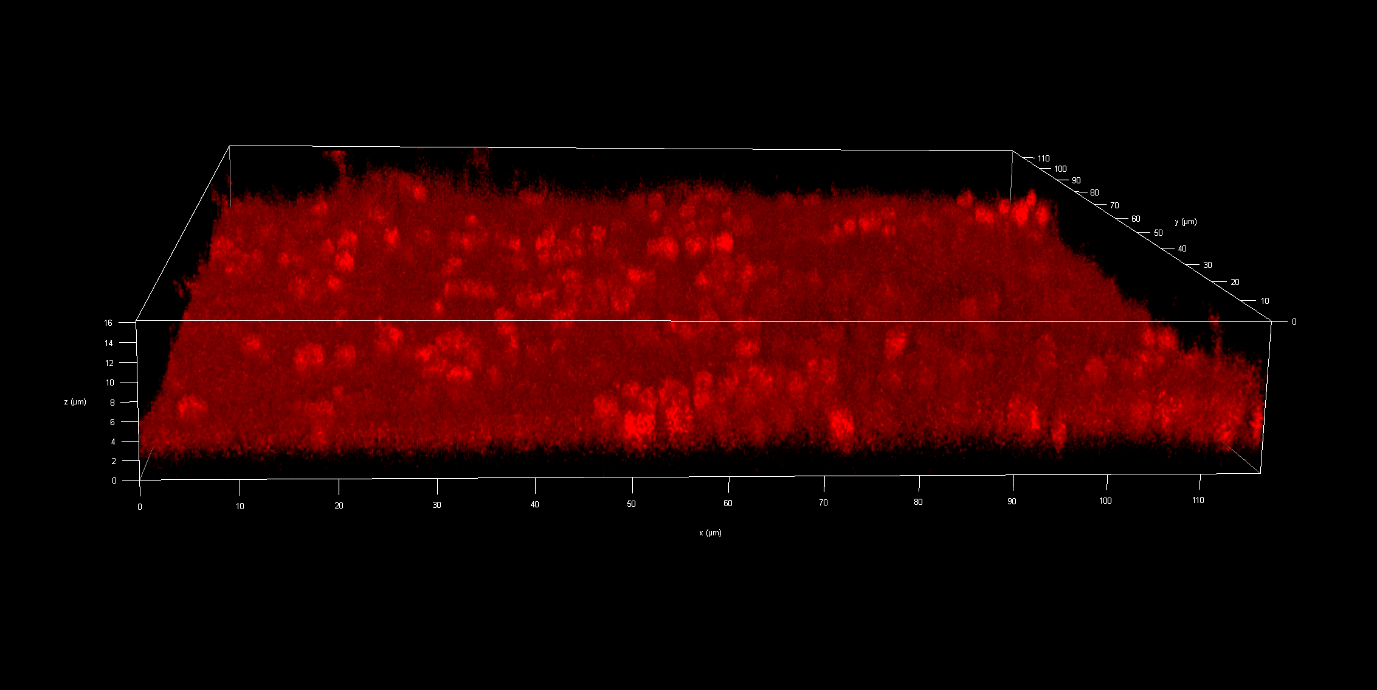
**

**Supp Fig 3. Confocal fluorescent microscopy of C. albicans 4 hour biofilm.**

To demonstrate the presence of an early biofilm containing extracellular matrix, chamber slides were stained with SYPRO™ Ruby Biofilm Matrix Stain following 4 hours incubation with *C. albicans* (see Supp Methods*)*. Image acquired using a Leica TCS SP8 confocal laser scanning microscope (Leica, UK). To preserve the image data (without modification) images were processed in 3D, using LAS-X software (Leica Application suite), for addition of a 3D scale.
