# Supplementary material for: Targeting Candida albicans in dual-species biofilms with antifungal treatment reduces Staphylococcus aureus and MRSA in vitro
Source: PLoS One. 2021 Apr 8;16(4):e0249547. doi: 10.1371/journal.pone.0249547 (PMC8031443; doi:10.1371/journal.pone.0249547)
Supplement: S1 Table — (DOCX) [file pone.0249547.s004.docx]

**Supplementary Table S1. qPCR Reaction formulation for *S. aureus*.**

| Component of reaction mixture | Volume for 1X12.5µl reaction | Final Concentration |
| --- | --- | --- |
| Platinum® qPCR Supermix-UDG X2 | 6.25 µl | 1X |
| Forward primer (200 µM):  CAAAGCATCCTAAAAAAGGTGTAGAGA | 0.025 µl | 400 nM |
| Reverse primer (200 µM):  TTCAATTTTCTTTGCATTTTCTACCA | 0.025 µl | 400 nM |
| Probe (100 µM):  6FAM-TTTTCGTAAATGCACTTGCTTCAGGACCA-BHQ1 | 0.025 µl | 200 nM |
| MgCl_2_ (50 mM) | 1.0 µl | 4mM |
| Template | 3 µl |  |
| Nuclease free water | 2.175 µl |  |
