# Supplementary material for: Targeting Candida albicans in dual-species biofilms with antifungal treatment reduces Staphylococcus aureus and MRSA in vitro
Source: PLoS One. 2021 Apr 8;16(4):e0249547. doi: 10.1371/journal.pone.0249547 (PMC8031443; doi:10.1371/journal.pone.0249547)
Supplement: S2 Table — (DOCX) [file pone.0249547.s005.docx]

**Table S2. qPCR Reaction formulation for *E. coli*.**

| Component of reaction mixture | Volume for 1X10µl reaction | Final Concentration |
| --- | --- | --- |
| FastStart Universal SYBR Green Master (2X) (Roche) | 5µl | 1X |
| Forward primer (200 µM):  AGAAGCTTGCTCTTTGCTGA | 0.025 µl | 500nM |
| Reverse primer (200 µM):  CTTTGGTCTTGCGACGTTAT | 0.025 µl | 500nM |
| Template | 1 µl |  |
| Nuclease free water | 3.95 µl |  |
