# Supplementary material for: Targeting Candida albicans in dual-species biofilms with antifungal treatment reduces Staphylococcus aureus and MRSA in vitro
Source: PLoS One. 2021 Apr 8;16(4):e0249547. doi: 10.1371/journal.pone.0249547 (PMC8031443; doi:10.1371/journal.pone.0249547)
Supplement: S3 Table — (DOCX) [file pone.0249547.s006.docx]

**Table S3. qPCR Reaction formulation for *P. aeruginosa*.**

| Component of reaction mixture | Volume for 1X12µl reaction | Final Concentration |
| --- | --- | --- |
| Platinum® qPCR Supermix-UDG | 6 µl | 1X |
| Forward primer (200 µM):  AACAGCGGTGCCGTTGAC | 0.024 µl | 400 nM |
| Reverse primer (200 µM):  GTCGGAGCTGTCGTACTCGAA | 0.024 µl | 400 nM |
| Probe (100 µM):  6FAM-TGAGCGACGAAGCC-MGB | 0.024 µl | 200 nM |
| MgCl_2_ (50 mM) | 0.24 | 4 mM |
| Template | 3 µl |  |
| Nuclease free water | 2.688 µl |  |
