# Supplementary material for: Targeting Candida albicans in dual-species biofilms with antifungal treatment reduces Staphylococcus aureus and MRSA in vitro
Source: PLoS One. 2021 Apr 8;16(4):e0249547. doi: 10.1371/journal.pone.0249547 (PMC8031443; doi:10.1371/journal.pone.0249547)
Supplement: S4 Table — (DOCX) [file pone.0249547.s007.docx]

**Table S4. qPCR Conditions for *C. albicans* and *E. coli* (instructions provided with FastSart kit, Roche).**

| Cycles | Target temperature | Hold time | Analysis Mode |
| --- | --- | --- | --- |
| 1 | 50ºC | 15 min | None |
| 1 | 95ºC | 10 min | None |
| 45 | 95ºC | 15 s | None |
|  | 60ºC | 60 s | Single fluorescence acquisition |
| Melting analysis | | | |
| 1 | 95ºC | 30 s | None |
| 1 | 35ºC | 60 s | None |
| 1 | 98ºC | 30 s | Continuous fluorescence acquisition |
| Cooling |  |  |  |
| 1 | 40ºC | 10 s | None |
