# Supplementary material for: Targeting Candida albicans in dual-species biofilms with antifungal treatment reduces Staphylococcus aureus and MRSA in vitro
Source: PLoS One. 2021 Apr 8;16(4):e0249547. doi: 10.1371/journal.pone.0249547 (PMC8031443; doi:10.1371/journal.pone.0249547)
Supplement: S5 Table — (DOCX) [file pone.0249547.s008.docx]

**Table S5. qPCR Conditions for *S. aureus* and *P. aeruginosa* (adapted from instructions provided with Platinum® Quantitative PCR SuperMix-UDG).**

| Cycles | Target temperature | Hold time | Analysis Mode |
| --- | --- | --- | --- |
| 1 | 50ºC | 15 min | None |
| 1 | 95ºC | 5 min | None |
| 45 | 95ºC | 10 s | None |
|  | 60ºC | 60 s | Single fluorescence acquisition |
| Cooling | | | |
| 1 | 40ºC | 10 s | None |
