# Supplementary material for: Targeting Candida albicans in dual-species biofilms with antifungal treatment reduces Staphylococcus aureus and MRSA in vitro
Source: PLoS One. 2021 Apr 8;16(4):e0249547. doi: 10.1371/journal.pone.0249547 (PMC8031443; doi:10.1371/journal.pone.0249547)
Supplement: S1 Methods — (DOCX) [file pone.0249547.s009.docx]

**Supporting Methods:**

Overnight cultures of *C. albicans* were prepared as outlined in the main manuscript and biofilms were formed in µ-Slide 8 well ibiTreat chamber slides (ibidi, Germany) by inoculating wells with 100 $\mu$l *C. albicans* (1.0 x 10^6^ cells/ml in RPMI). Wells were incubated for 4 hours to allow initial *C. albicans* biofilm formation. Slides were and then incubated with SYPRO™ Ruby Biofilm Matrix Stain (Invitrogen Molecular Probes) for 30 min. Images were acquired using a Leica TCS SP8 confocal laser scanning microscope (Leica UK). To preserve the image data (without modification) images were processed in 3D using LAS-X software (Leica Application suite), for addition of a 3D scale.
